# Supplementary material for: Characterization a Novel Butyric Acid-Producing Bacterium Collinsella aerofaciens Subsp. Shenzhenensis Subsp. Nov
Source: Microorganisms. 2019 Mar 13;7(3):78. doi: 10.3390/microorganisms7030078 (PMC6463082; doi:10.3390/microorganisms7030078)
Supplement: Supplementary file 1 [file microorganisms-07-00078-s001.pdf]

Table S1: The effect of temperature, pH, NaCl and bile on growth of TF06-26 and *C. aerofaciens* ATCC 25986^T

		TF06-26	*C. aerofaciens* ATCC 25986^T
Temperature (°C)	10	−	−
	15	−	−
	20	−	−
	25	+	+
	30	+	+
	35	+	+
	37	++	++
	42	+	+
	45	+	+
	50	−	−
pH	3	−	−
	3.5	−	−
	4	−	−
	4.5	−	−
	5	+	−
	5.5	+	−
	6	+	+
	6.5	+	+
	7	++	++
	7.5	+	++
	8	+	+
	8.5	−	w
	9	−	−
	10	−	−
NaCl % (w/v)	0	+	+
	0.005	+	+
	0.01	+	+
	0.02	+	+
	0.03	−	−
	0.04	−	−
	0.05	−	−
	0.06	−	−
	0.07	−	−
Bile % (w/v)	0	+	+
	0.05	+	+
	0.1	+	+
	0.2	+	w
	0.3	w	w
	0.4	−	−

	0.5	—	—

[T]: Type strain.

Table S2: The antibiotic susceptibility test results of TF06-26

	Zone of Inhibition(mm)
Ampicillin	2.8
Carbenicillin	2.8
Cefazolin	2.7
Penicillin	2.6
Cephalexin	2.6
Piperacillin	2.6
Cefuroxime	2.4
Ceftriaxone sodium	2.4
Cefoperazone	2.4
Tetracycline	2.2
Cephradine	2.0
Ceftazidime	1.8
Oxacillin	1.5
Gentamicin	0.8
Kanamycin	0
Amikacin	0
Framycetin	0

[T]: Type strain.

Table S3: The GenBank accession, total length (bp) and GC content (%) of 13 strains of genus *Collinsella*

	GenBank accession	Total length (Mb)	GC content (%)
C. aerofaciens ATCC 25986^T	NZ_AAVN00000000.2	2.44	60.55
C. bouchesdurhonensis Marseille-P3296 ^{T}	NZ_FTLD00000000.1	1.88	57.94
C. intestinalis DSM 13280^T	NZ_ABXH00000000.2	1.81	62.47
C. phocaeensis Marseille-P3245^T	NZ_FQLR00000000.1	2.11	65.50
C. stercoris DSM 13279^T	NZ_ABXJ00000000.1	2.48	63.19
C. tanakaei YIT 12063^T	NZ_ADLS00000000.1	2.50	60.24
C. ihuae GD8 ^{T}	NZ_FCOU00000000.1	2.84	64.13
C. provencensis Marseille-P3740 ^{T}	NZ_FZRI00000000.1	1.74	58.21
C. vaginalis Marseille-P2666^T	NZ_FWYK00000000.1	2.14	64.57
C. aerofaciens 2789STDY5834902	NZ_CZAQ00000000.1	2.19	60.10
C. aerofaciens indica	NZ_CP024160.1	2.31	60.10
C. aerofaciens 2789STDY5608842	NZ_CYYF00000000.1	2.14	60.00
C. aerofaciens 2789STDY5608823	NZ_CYYP00000000.1	2.23	59.60

^{T}: Type strain.

Table S4: The ANIb values between TF06-26 and 5strain of species *C. aerofaciens*

	1	2	3	4	5	6
1	-	93.90	94.08	93.22	93.05	92.92
2	93.90	-	93.44	93.18	93.12	93.07
3	94.08	93.44	-	93.73	93.60	93.49
4	93.22	93.18	93.73	-	94.93	94.77
5	93.05	93.12	93.60	94.93	-	94.32
6	92.92	93.07	93.49	94.77	94.32	-

1, TF06-26; 2, *C. aerofaciens* 2789STDY5834902; 3, *C. aerofaciens* indica; 4, *C. aerofaciens* 2789STDY5608842; 5, *C. aerofaciens* 2789STDY5608823; 6, *C. aerofaciens* ATCC 25986^T;
^{T}: Type strain.

Table S5: The core gene, accessory genes and unique genes number of TF06-26 and 13 strains of genus *Collinsella*

Organism name	Core genes	Accessory genes	Unique genes
C. bouchesdurhonensis Marseille-P3296 ^{T}	535	719	331
C. intestinalis DSM 13280^{T}	535	767	200
C. phocaeensis Marseille-P3245^{T}	535	816	296
C. stercoris DSM 13279^{T}	535	871	435
C. tanakaei YIT 12063^{T}	535	968	583
C. ihuae GD8 ^{T}	535	845	824
C. provencensis Marseille-P3740 ^{T}	535	533	347
C. vaginalis Marseille-P2666^{T}	535	596	517
TF06-26	535	1206	130
C. aerofaciens ATCC 25986^{T}	535	1253	135
C. aerofaciens 2789STDY5834902	535	1145	82
C. aerofaciens indica	535	1216	103
C. aerofaciens 2789STDY5608842	535	1150	79
C. aerofaciens 2789STDY5608823	535	1201	98

^{T}: Type strain.

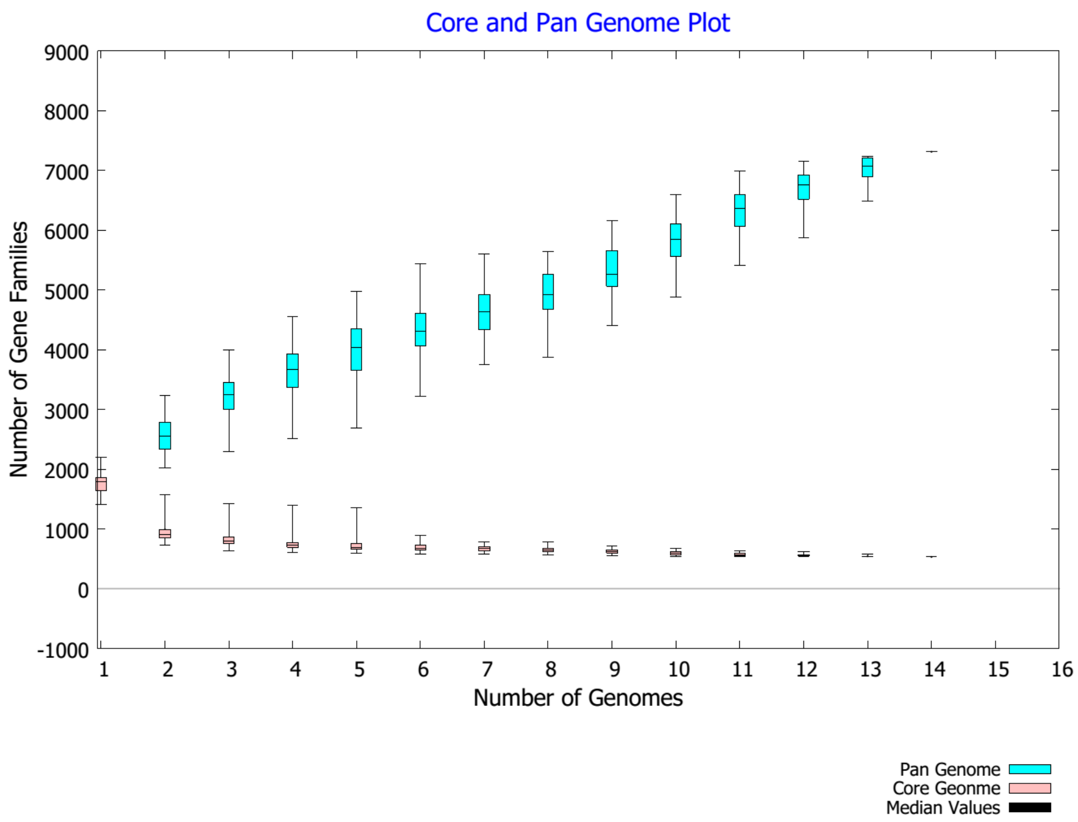

Figure S1. The core genome and pan genome of TF06-26 and13 genomes of the genus *Collinsella.*

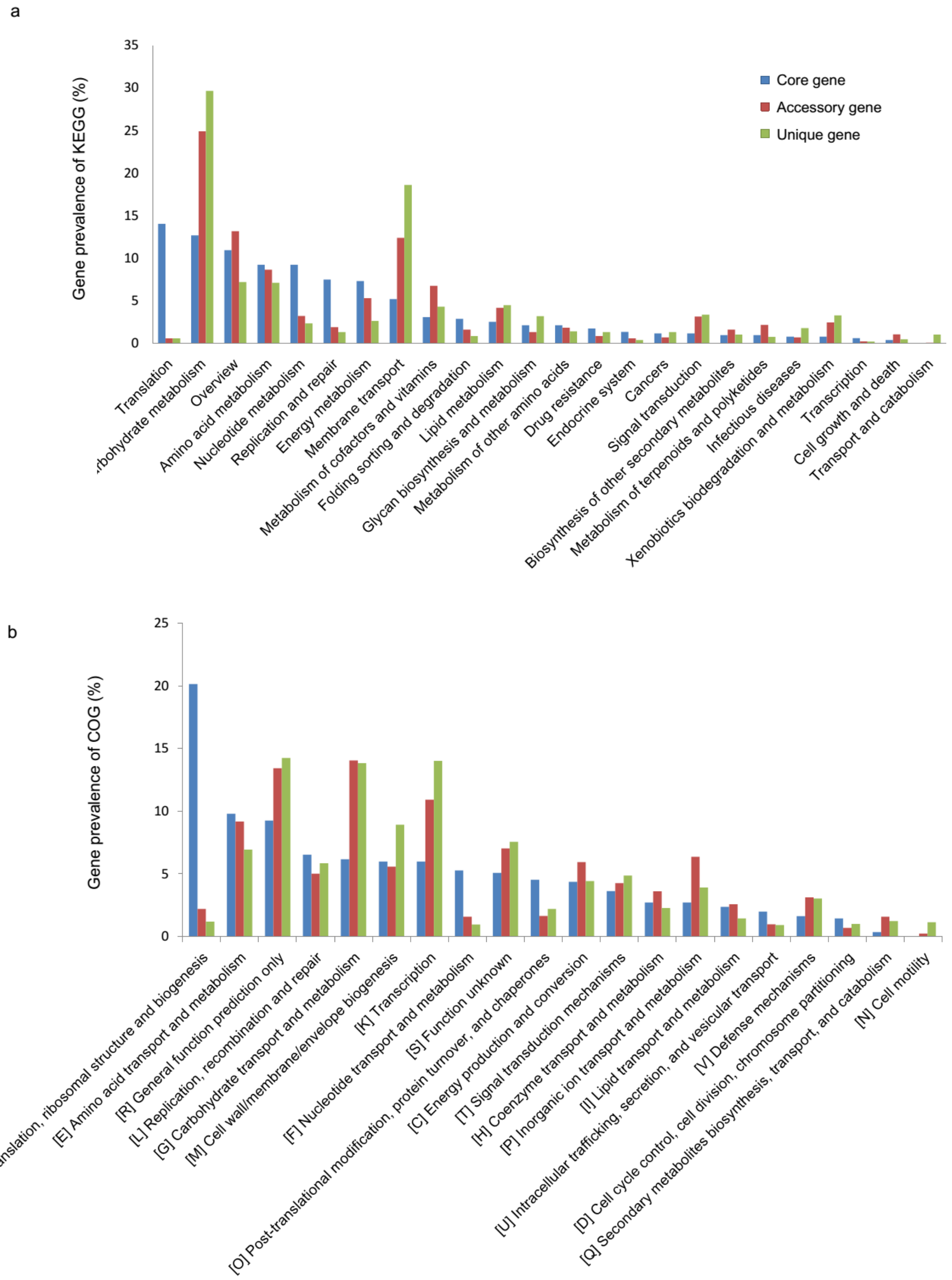

Figure S2. Function annotation of the core, accessary and unique genes of 14 genomes. (a) KEGG-based functional annotation; (b) COG-based functional classification.
